# The psychosocial burden of survivorship: long-term health-related quality of life following Fournier's gangrene

**DOI:** 10.3389/fsurg.2026.1910161

**Published:** 2026-08-04

**Authors:** Berk Goktepe, Safa Vatansever, Kamil Erozkan, Recep Temel, Osman Bozbiyik, Cemil Caliskan, Erhan Akgun, Mustafa Ali Korkut

**Affiliations:** Department of General Surgery, Ege University Hospital, Izmir, Türkiye

**Keywords:** Fournier's gangrene, Fournier's Gangrene Severity Index, health-related quality of life, SF-36, survivorship

## Abstract

**Background:**

Fournier's gangrene (FG) is a life-threatening necrotizing fasciitis of the perineal and genital regions associated with high morbidity and mortality. While the Fournier's Gangrene Severity Index (FGSI) effectively predicts acute mortality, its utility in prognosticating long-term health-related quality of life (HRQoL) remains unclear. We aim to evaluate the long-term HRQoL of FG survivors using age-standardized norms, assess the predictive value of admission FGSI on long-term functional outcomes, and determine the impact of postoperative recovery duration on survivorship.

**Methods:**

This retrospective cross-sectional study evaluated adult FG survivors treated at a tertiary academic center over 14 years. HRQoL was assessed using the 36-Item Short Form Health Survey (SF-36). Raw scores were transformed into age-adjusted Z-scores using validated local normative data. Primary outcomes were deviations in Z-scores, Physical Component Summary (PCS), and Mental Component Summary (MCS) scores from normative baselines. Secondary outcomes evaluated the correlation of SF-36 domains with admission FGSI and time elapsed since index surgery.

**Results:**

The cohort comprised 36 long-term survivors. Survivors exhibited profound impairments in HRQoL, significantly deviating from age-matched norms across multiple domains, most notably in social functioning and emotional roles. Both mean (SD) scores of PCS [44.9 (12.9) vs. 52.6 (8.8), *p* = 0.001] and MCS [32.4 (13.8) vs. 51.7 (5.7), *p* < 0.001] were significantly lower than the general population norms. Admission FGSI demonstrated no statistical correlation with any long-term SF-36 domain (all *p* > 0.05). Conversely, increased time since surgery positively correlated with continuous improvements in physical functioning, social functioning, emotional roles, vitality, and bodily pain (all *p* < 0.05).

**Conclusion:**

FGSI does not appear to predict long-term HRQoL in survivors of FG, suggesting a dissociation between acute disease severity and survivorship outcomes. While FG inflicts severe, predominantly psychosocial morbidity, patient outcomes demonstrate significant temporal improvement. These findings emphasize the need for patient-centered outcome measures beyond acute prognostic scoring systems.

## Introduction

Fournier's gangrene (FG) is a rapidly progressive, highly lethal polymicrobial necrotizing fasciitis affecting the fascial planes of the perineal, perianal, and genital regions (1, 2). Historically, the clinical paradigm surrounding FG has been overwhelmingly “mortality-centric,” focusing on aggressive surgical debridement, broad-spectrum antimicrobial therapy, and short-term survival (3). Modern advancements in critical care and reconstructive surgery have successfully expanded the cohort of patients surviving the acute physiological storm of this disease (4). Consequently, the clinical imperative must now shift toward understanding the multi-year trajectory of physical, functional, and psychological recovery.

The Fournier's Gangrene Severity Index (FGSI) has served as the gold-standard prognostic tool for decades, utilizing admission physiological parameters to accurately predict acute mortality (5). It was not designed to capture the profound anatomical disfigurement, the necessity for repeated extensive surgical debridements, or the debilitating presence of fecal diversions that characterize the aftermath of FG (2).

Despite the critical importance of holistic patient recovery, comprehensive empirical evaluations of long-term health-related quality of life (HRQoL) in FG survivors remain exceptionally rare. Existing literature frequently relies on raw HRQoL scores without appropriate age-matched control groups, rendering it difficult to delineate disease-specific functional deficits from expected age-related physiological decline (3, 6, 7). Furthermore, the relationship between acute physiological presentation (FGSI) and longitudinal functional recovery has not been rigorously investigated. Addressing these gaps requires an instrument capable of capturing the diverse dimensions of patient well-being, as FG patients face both profound physical trauma and significant psychosocial distress due to perineal and genital disfigurement.

The primary aim of this study was to conduct an exhaustive evaluation of the long-term HRQoL of FG survivors by utilizing age-standardized Z-scores derived from highly validated local normative data (8). To capture both the physical and psychological toll of the disease, we specifically employed the Medical Outcomes Study 36-Item Short Form Health Survey (SF-36), which provides a well-validated, multi-dimensional profile of health status and exhibits high sensitivity to the distinct domains of post-surgical functional recovery (9). We hypothesized that FG survivors experience profoundly diminished HRQoL compared to age-matched norms, particularly in psychosocial domains.

## Materials and methods

### Study design and patient selection

This single-center, retrospective, cohort study was conducted in strict adherence to the Strengthening the Reporting of Observational Studies in Epidemiology (STROBE) guidelines (10). The protocol was approved by the Institutional Review Board (Approval Number: 24-9T/36) and conducted in accordance with the Declaration of Helsinki (11). Informed consent was obtained from all participants.

The study included a consecutive cohort of patients who underwent surgical treatment for FG between January 2014 and December 2024 at a tertiary referral center. Eligible patients were adults aged 18 years or older at the time of diagnosis with a surgically and microbiologically confirmed diagnosis of FG, defined as necrotizing fasciitis involving the perineal and/or genital fascial planes. Only patients who survived beyond the index hospitalization and were therefore eligible for long-term survivorship assessment were included. Participants were also required to have sufficient cognitive and linguistic capacity to independently complete the psychometric survey. Patients were excluded if they died during the index admission or before survey administration. Cases limited to localized perianal abscesses without evidence of fascial necrosis were not considered FG and were therefore excluded. Patients with missing acute clinical records that precluded accurate calculation of the Fournier's Gangrene Severity Index (FGSI) were also excluded. In addition, individuals with severe pre-existing psychiatric disorders or terminal medical conditions that were considered likely to substantially alter baseline health-related quality of life were excluded from the analysis.

### Clinical parameters and outcomes

Disease severity at admission was calculated using the FGSI, derived from the most aberrant physiological parameters within the first 24 h (1, 5). Systemic disease burden was stratified using the Charlson Comorbidity Index (CCI) (12). The primary psychometric instrument utilized was the SF-36 (9). The SF-36 evaluates eight domains: Physical Functioning (PF), Role-Physical (RP), Bodily Pain (BP), General Health (GH), Vitality (VT), Social Functioning (SF), Role-Emotional (RE), and Mental Health (MH). The SF-36 health survey was administered at a single time point during each patient's final follow-up evaluation. “Follow-up duration” and “time since surgery” are synonymous and represent the exact same temporal interval in our analysis, spanning from the date of the initial emergency surgery to the date of HRQoL questionnaire administration.

The primary outcome was the quantifiable divergence in long-term HRQoL between the FG survivor cohort and the healthy normative population. To achieve this, raw SF-36 scores were mathematically transformed into age-adjusted Z-scores utilizing comprehensive, locally validated epidemiological normative data from the Izmir urban population (8). Z-scores were calculated as follows: Z-score = (Patient score – *μ*_age group_)/σ_age group_. Z-score of 0 represents the normative population mean, while negative values indicate HRQoL below the age-matched norm. To determine whether SF-36 Z-scores differed significantly from the normative population, one-sample t-tests were applied, using zero as the reference value. Additionally, Physical Component Summary (PCS) and Mental Component Summary (MCS) scores were derived using orthogonal algorithms and compared against national population norms (13).

Secondary outcomes evaluated the statistical correlation between long-term SF-36 domain scores and acute disease severity (FGSI), temporal duration elapsed since index surgery, and major clinical covariates [e.g., stoma formation, vacuum-assisted closure (VAC) therapy].

### Statistical analysis

Analyses were performed using IBM SPSS Statistics for Windows, version 25.0 (Armonk, NY). To prevent imputation bias, a complete case analysis methodology was applied; only fully completed SF-36 questionnaires were processed. Survivor bias was handled inherently by strictly bounding inferential claims to the survivorship cohort, avoiding inappropriate extrapolation to acute non-survivors. Normality was assessed via the Shapiro–Wilk test. Continuous variables were expressed as mean (SD) or median (range). Categorical variables were presented as frequencies and percentages. One-sample t-tests evaluated primary Z-score and summary score deviations against a zero-baseline. Two-group comparisons utilized the independent Student's t-test or Mann–Whitney U test based on distribution. Bivariate associations were assessed using Spearman rank-order correlation matrices. A two-tailed *p*-value of <0.05 determined statistical significance.

## Results

**Figure 1:**
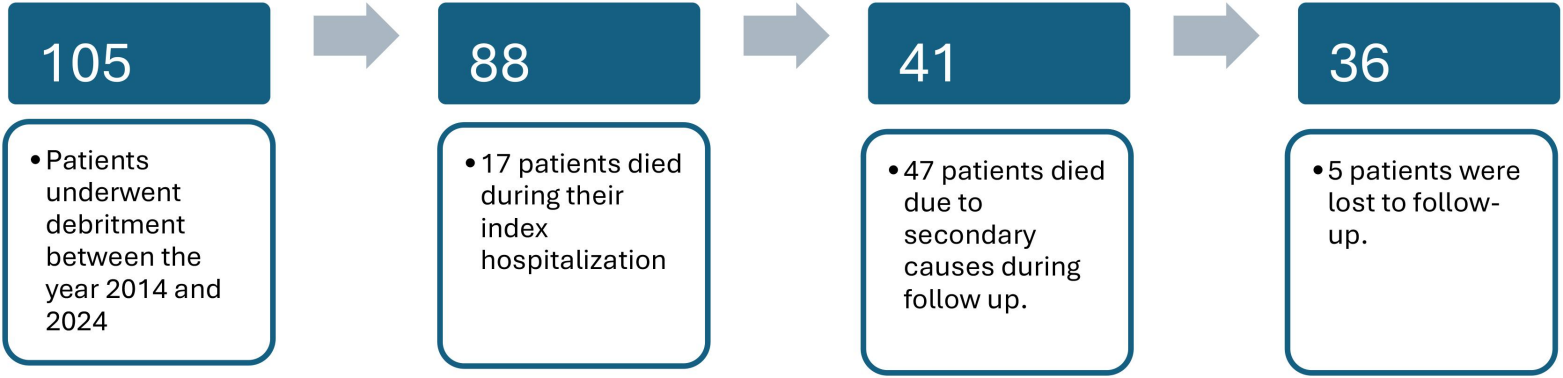
Diagram for patient selection.

From an initial consecutive cohort of 105 FG patients, acute in-hospital mortality was 16.2% (*n* = 17). During the multi-year follow-up, 47 (44.8%) additional patients died to secondary etiologies, leaving a cohort of 41 (39.0%) long-term survivors. Within this group of 41 survivors, 5 patients (12.2%) were lost to follow-up and excluded. Consequently, the remaining 36 out of 41 long-term survivors (87.8%) completed the SF-36 questionnaire and were included in the final analysis (Figure 1).

**Figure 2:**
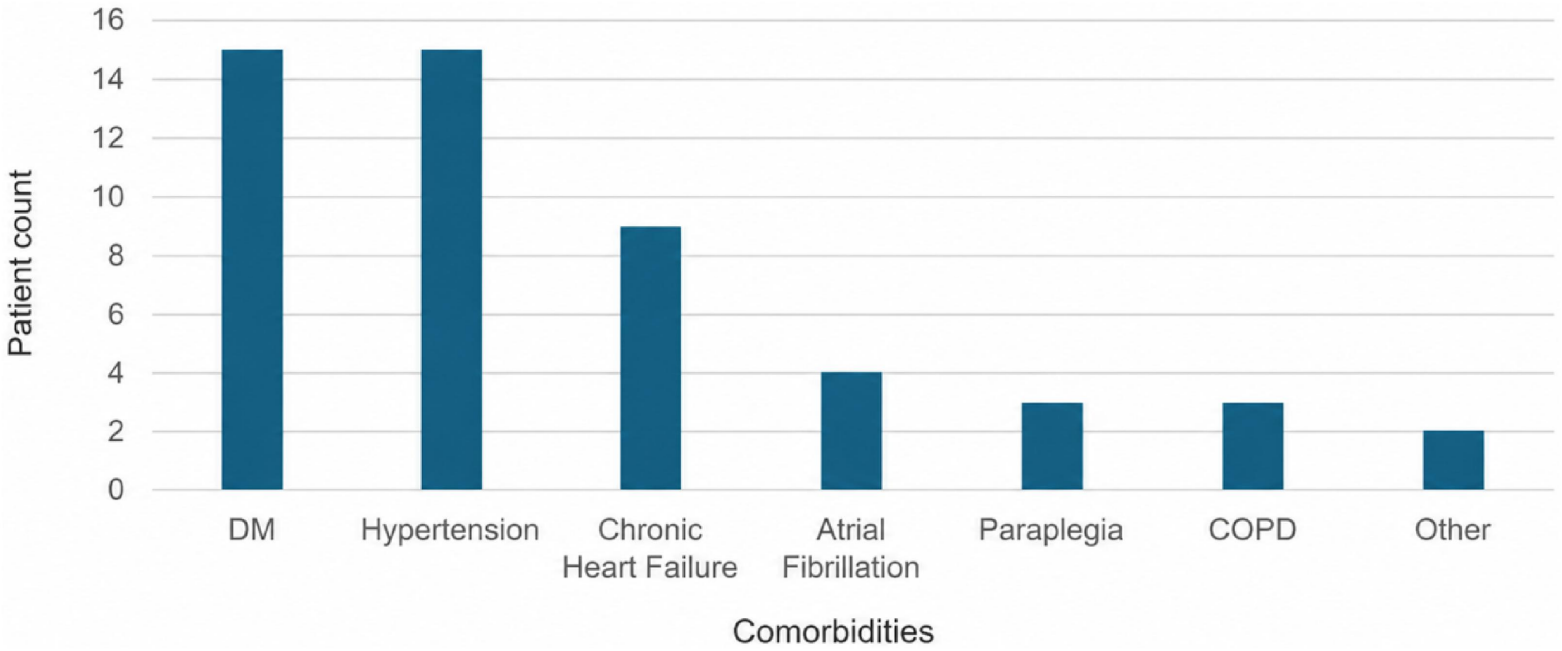
Comorbidities.

The cohort consisted of 28 men (77.8%) and 8 women (22.2%), with a mean age of 54 (10.9) years. Median longitudinal follow-up was 53.5 months (range: 1–122). The pre-morbid disease burden was substantial: 24 patients (66.7%) possessed at least one major comorbidity (median CCI: 2.5), primarily hypertension (*n* = 15, 41.7%) and diabetes mellitus (*n* = 15, 41.7%) (Figure 2).

Surgical burden was extensive: 20 patients (55.6%) required sequential re-debridements (totaling 83 procedures), 24 patients (66.7%) required VAC therapy, and 8 patients (22.2%) underwent stoma formation. The median admission FGSI was 3.0 (range: 0–10) (1). Admission FGSI scores demonstrated no association with clinical demographics or surgical requirements (Table 1).

**Table 1:** Comparison of FGSI scores across clinical parameters.

Clinical parameter	Median FGSI (range)	*p* value*
Gender		0.302
Male (*n* = 28)	3.0 (0–10)	
Female (*n* = 8)	4.0 (2–8)	
Comorbidity		0.311
Absent (*n* = 12)	2.0 (0–6)	
At least one (*n* = 24)	3.5 (0–10)	
Additional Debridement		0.236
Absent (*n* = 16)	2.0 (0–7)	
At least one (*n* = 20)	3.5 (0–10)	
VAC Utilization		0.128
No (*n* = 12)	2.0 (1–6)	
Yes (*n* = 24)	3.5 (0–10)	
Stoma		0.641
Absent (*n* = 28)	2.0 (0–10)	
Present (*n* = 8)	4.0 (0–8)	

FGSI, Fournier's Gangrene Severity Index.

* Mann–Whitney U test.

**Figure 3:**
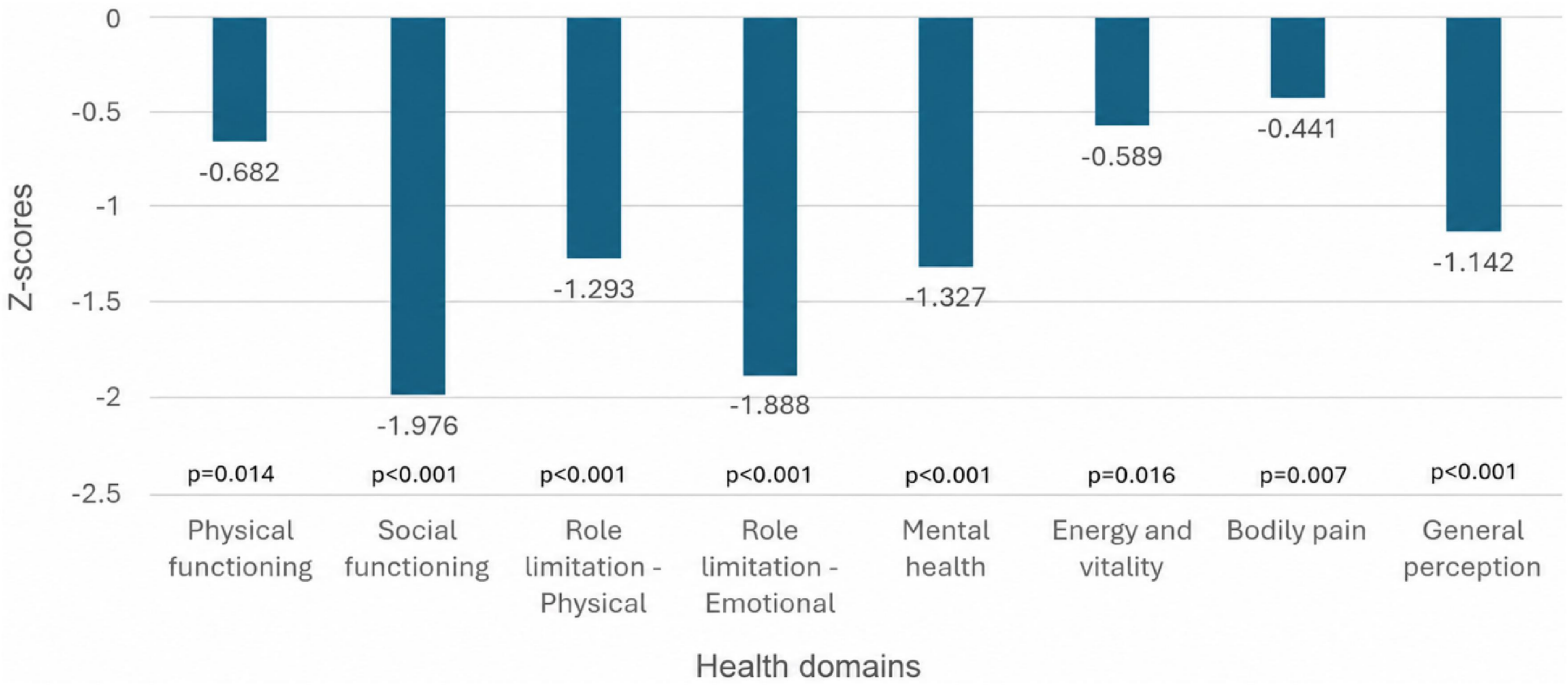
Age-standardized mean Z-scores of health-related quality of life (SF-36) domains. The zero baseline represents the standardized general population Z-score mean (*μ* = 0, σ = 1). Negative values indicate poorer quality of life, and the values on the bars represent the cohort's average deviation from the normative population. The *p*-values denote the statistical significance of the deviation from the population norm, analyzed via a one-sample t-test.

Age-adjusted Z-score transformations revealed profound, statistically significant decrements across the HRQoL spectrum compared to the normative population (Figure 3). The most severe deviations were mapped to psychosocial domains, specifically SF and RE. Moderate impairments were recorded in MH, RP, and GH. Macro-level summary scores confirmed this global deterioration. The survivor cohort exhibited a severely compromised mean PCS of 44.9 (12.9) compared to the national norm of 52.6 (8.8) (*p* = 0.001). The psychological detriment was even more pronounced, with the mean MCS plummeting to 32.4 (13.8) against a norm of 51.7 (5.7) (*p* < 0.001).

Extensive cross-tabulation evaluated the impact of clinical parameters on raw SF-36 scores (Table 2). Notably, surgical morbidity factors, including the presence of a stoma, necessity for multiple debridements, or VAC application, did not independently alter long-term HRQoL (all *p* > 0.05). However, pre-morbid baseline health significantly dictated outcomes; patients with pre-existing comorbidities exhibited significantly depressed SF scores (*p* = 0.041) and exacerbated BP scores (*p* = 0.012).

**Table 2:** Comparison of SF-36 scores across clinical parameters.

Parameters	PF*	RP*	RE*	VT*	MH*	SF*	BP*	GH*
Gender								
Male (*n* = 28)	82.5	25.0	50.0	60.0	56.0	62.5	72.5	57.5
Female (*n* = 8)	70.0	25.0	83.4	52.5	58.0	62.5	72.5	40.0
*p* value**	0.723	0.641	0.808	0.641	0.926	0.808	0.955	0.985
Comorbidity								
Absent (*n* = 12)	80.0	87.5	100	60.0	60.0	81.3	80.0	67.5
At least one (*n* = 24)	77.5	0.00	0.00	52.5	54.0	62.5	60.0	40.0
*p* value**	0.416	0.171	0.349	0.497	0.655	0.041	0.012	0.090
Additional Debridement								
Absent (*n* = 16)	87.5	12.5	83.4	57.5	60.0	68.8	78.7	57.5
At least one (*n* = 20)	72.5	37.5	50.0	57.5	54.0	62.5	67.5	52.5
*p* value**	0.560	0.741	0.888	0.765	0.765	0.789	0.962	0.912
Stoma								
Absent (*n* = 28)	80.0	12.5	83.5	55.0	54.0	62.5	77.5	52.5
Present (*n* = 8)	70.0	62.5	50.0	60.0	58.0	62.5	61.5	55.0
*p* value**	0.537	0.614	0.955	0.537	0.751	0.284	0.284	0.926
Complication								
Absent (*n* = 32)	77.5	25.0	83.3	57.5	58.0	62.5	77.5	57.5
Present (*n* = 4)	47.5	50.0	50.0	57.5	48.0	43.8	55.0	35.0
*p* value**	0.511	0.942	0.981	0.827	0.248	0.340	0.157	0.543

PF, physical functioning; RP, role limitations due to Physical health; RE, Role limitations due to Emotional problems; VT, vitality; MH, mental health; SF, social functioning; BP, bodily pain; GH, general health.

* Median values were reported.

** Mann–Whitney U test.

Critically, correlation matrices demonstrated that admission FGSI scores completely failed to predict long-term HRQoL. The FGSI yielded no statistically significant correlation with any of the eight SF-36 domains (Table 3). Conversely, the duration of postoperative convalescence emerged as a highly significant therapeutic modifier. Increased time elapsed since the index surgery positively and significantly correlated with improved PF (r = 0.448, *p* = 0.006), SF (r = 0.415, *p* = 0.012), RE (r = 0.338, *p* = 0.043), VT (r = 0.393, *p* = 0.018), and BP (r = 0.400, *p* = 0.016).

**Table 3:** Correlation between FGSI and SF-36 domains*.

SF-36 domain	r_s_ (FGSI)	*p*-value*
PF	0.193	0.258
RP	0.121	0.483
RE	0.191	0.264
VT	0.219	0.200
MH	0.203	0.235
SF	0.220	0.198
BP	0.123	0.475
GH	0.189	0.269

SF-36, Short form 36; FGSI, Fournier's Gangrene Severity Index; PF, physical functioning; RP, role limitations due to Physical health; RE, role limitations due to Emotional problems; VT, vitality; MH, mental health; SF, social functioning; BP, bodily pain; GH, general health.

* Spearman's correlation analysis.

## Discussion

This analysis establishes that FG survivors endure profound, multi-domain HRQoL impairments long after surgical wounds have healed. Utilizing local normative data to control for age-related decline, the data unequivocally isolates the disease's devastating toll. Strikingly, the most severe deficits observed were not localized to somatic debility, but rather permeated the psychosocial domains (SF and RE). While physical functioning was demonstrably compromised (PCS: 44.9 vs. 52.6), the mental health devastation was critically disproportionate (MCS: 32.4 vs. 51.7).

These findings illuminate the often-invisible sequelae of massive urogenital and perineal tissue loss. The ensuing disfigurement, potential for stoma placement, and chronic neuropathic pain act as catalysts for profound psychological distress (4). The drastic reductions in social reintegration strongly suggest complex interplays of persistent stigma, altered body schema, and sexual dysfunction, corroborating contemporary multi-center reconstructive data indicating that sexual health and social functioning remain stubbornly refractory to surgical correction alone (6, 14). Similarly, our study confirmed persistent impairment in physical health; however, the most prominent deficits in our cohort were observed in psychosocial domains. This discrepancy may be related to cultural differences, differences in follow-up duration, or the use of age-standardized normative comparisons in our study.

FG remains a life-threatening condition associated with considerable mortality. In our cohort, the in-hospital mortality rate was 16.2%, which is consistent with previously reported rates ranging between 16% and 40% in contemporary series (3, 15). Moreover, an additional 44.7% of patients died during long-term follow-up, reflecting the severe systemic burden and high comorbidity profile of this population. Therefore, the current cohort represents a selected group of long-term survivors, and the true overall burden of the disease may be even greater than our findings suggest. Survivor bias is an important limitation in the interpretation of long-term HRQoL outcomes in this setting.

When evaluating the HRQoL outcomes across clinical subgroups, our results revealed that the presence of comorbidities significantly deteriorated SF and BP domains. However, for the majority of the other clinical parameters, no statistically significant differences were observed across the SF-36 subscales. Although several SF-36 subscales demonstrated considerable differences in median scores between these subgroups, these differences did not reach statistical significance. This finding is likely attributable to the limited sample size and the skewed distribution of the data (as indicated by our use of median values and non-parametric Mann–Whitney U tests), which reduced the statistical power to detect between-group differences. Therefore, these descriptive variations should be interpreted with caution and should not be overinterpreted as consistent clinical differences between the groups.

A pivotal, hypothesis-confirming discovery of this study is the absolute dissociation between acute physiological severity and long-term functional recovery. The FGSI is universally validated as an indispensable tool for predicting imminent acute mortality and guiding fluid resuscitation (5, 16). However, our correlation matrices demonstrate no significant association between admission FGSI scores and long-term HRQoL domains. Our findings support previous observations by Wang et al., who emphasized that although FGSI is a reliable prognostic indicator in the acute setting, long-term HRQoL after FG is strongly influenced by factors extending beyond initial physiological severity, including repeated debridements, intensive care burden, and comorbidity control (17). Therefore, “clinical success” must be radically redefined; achieving hospital discharge is merely the inception of a multi-year rehabilitative marathon.

In stark contrast to the FGSI, the duration of the postoperative recovery period was a powerful positive predictor of HRQoL. The robust correlations between temporal distance from surgery and improved functional, social, and pain domains indicate that holistic recovery is a highly dynamic, protracted, but ultimately positive process. This reflects the necessary biological time for massive wound maturation, scar remodeling, and complex psychological adaptation to an altered body schema (2, 3, 17). However, this rehabilitative trajectory is severely compounded by systemic pre-morbidities. While aggressive surgical interventions (e.g., VAC or stomas) did not independently depress long-term outcomes, the sheer presence of baseline metabolic or vascular comorbidities (predominantly DM and hypertension) significantly limited social functioning and exacerbated chronic pain. The profound physiological stress of necrotizing sepsis further destabilizes these chronically ill patients, rendering multidisciplinary comorbidity control critical to functional recovery (3).

This study exhibits robust methodological strengths that satisfy rigorous epidemiological reporting standards. It utilizes mathematically precise, age-standardized Z-scores derived directly from local normative data, explicitly controlling for baseline population variables (8). Dual calculation of PCS/MCS against broader national norms provides an unassailable metric of absolute deficit (13). The retrospective, single-center design necessitates a modest absolute sample size (*n* = 36, representing 34.3% of the initial 105-patient cohort); however, given the extreme rarity of the disease, the massive acute mortality rate, and the strict 14-year inclusion framework, this cohort provides high statistical power for a single-institution series. Concurrently, due to this modest sample size, a multivariate analysis could not be safely performed to adjust for potential confounding factors, as building multivariate models with a limited number of subjects risks over-fitting and statistical instability. Similarly, certain clinical variables, such as additional surgical debridements, were analyzed dichotomously rather than quantitatively. While this binary classification was statistically necessary to maintain group sizes and prevent model instability in our modest cohort, we acknowledge that it may oversimplify the clinical spectrum of surgical intensity, representing a limitation of our study design. Furthermore, while inherent survivor bias exists due to the high mortality rate (precluding analysis of the most severely afflicted incident cases), this is an unavoidable, defining condition of survivorship research. The explicit objective was to characterize the functional plateau of survivors; therefore, this bias appropriately focuses the inferential scope. Although the 36 included patients had entirely complete data with zero missing questionnaire items, the fact that 5 long-term survivors (12.2%) were lost to follow-up introduces a potential attrition bias, as those lost to follow-up patients might harbor distinct, unmeasured clinical characteristics. Finally, while the use of validated normative controls rigorously mitigated the absence of pre-morbid baseline data, this unavoidable reality of emergency surgery constitutes a structural limitation that could not be entirely eliminated.

## Conclusion

Survivors of FG endure profound, multi-systemic HRQoL impairments that heavily disproportionately afflict the psychosocial and emotional domains. A critical prognostic gap exists: acute physiological scoring systems (such as the FGSI) may not adequately reflect the long-term quality of the life saved and show limited prognostic utility for predicting longitudinal recovery in modest cohorts. However, survivorship demonstrates significant, quantifiable amelioration over time. Management paradigms must evolve beyond binary acute survival to systematically integrate early psychological screening, sexual rehabilitation, and long-term multidisciplinary follow-up pathways.

## Data Availability

The raw data supporting the conclusions of this article will be made available by the authors, without undue reservation.

## Author's note

This study was presented as an oral podium presentation at the 20th National and 3rd International Turkish Colon and Rectal Surgery Congress, May 16–20, 2025, Antalya-Turkiye
